# Comparative case-study of implementation of a coordinated rehabilitation care pathway between municipalities and hospitals for stroke patients in the Central Denmark region

**DOI:** 10.1186/1472-6963-14-S2-O12

**Published:** 2014-07-07

**Authors:** Karla Douw

**Affiliations:** 1 Centre for Public Health & Quality Improvement, Aarhus, Denmark

## Background

In May 2012, the health authority (RHA) of the Central Denmark Region decided to transfer inpatient rehabilitation for stroke patients to community-based rehabilitation, as part of a major reform of regional stroke care. It was implemented top-down in three months. Patients were promised a more integrated care pathway with early discharge stroke teams bridging the two sectors. In this decentralized system, hospital care is the responsibility of the Regional health authority, and rehabilitation at home that of municipalities. The implementation of this reform therefore poses some challenges. The HA’s reform required written cooperation agreements to be made by hospitals and municipalities. The implementation of these agreements and their aim of establishing a coordinated rehabilitation pathway was analysed, with a focus on (inter) organisational aspects, as the change was similar throughout the region.

## Materials and methods

A multiple case study was designed, which compared cases of municipalities (n=7), and cases of stroke teams (n=5). Municipalities were selected based on two criteria from Greenhalgh’s conceptual model for determinants of implementation of innovations: size (proxy for slack resources and professionalization) and the existence of a boundary spanner in the implementation process. We hypothesized that these factors influence the implementation of the cooperation agreements. Data was gathered by means of semi-structured interviews (n=12), and document analysis.

## Results

Both sectors accepted the change, as they believe the reform will benefit the patient. The study does not show any influence of size of the municipality, and having a boundary spanner in the process, on the implementation of coordination of stroke rehabilitation care between stroke teams and municipalities. Stroke teams experience opposition of municipalities to their existence, as municipalities state they do not need the Region’s stroke teams, who they are obliged to reimburse for their services. Stroke teams feel they coordinate care with municipalities. Municipalities do not experience this. Care is therefore not optimally coordinated around the patient. Ease-of-access to contact-persons is the main challenge for coordination at practice level.

## Conclusion

The cooperation agreements might have been implemented at the administrative level, but at the practice level the coordination of care is lacking. Facilitating regular face-to-face contact between both sectors seems crucial for further implementation at practice level. In the future, financial consequences need to be communicated before implementation, and top-down implementation needs to be complemented with local implementation plans at lower levels of health care, with a clearer role for the boundary spanners.

